# Analyzing the Behavior of Cannabis Users during the COVID-19 Confinement in Spain

**DOI:** 10.3390/ijerph182111324

**Published:** 2021-10-28

**Authors:** Sergio Fernández-Artamendi, Manuel J. Ruiz, Carla López-Núñez

**Affiliations:** 1 Department of Psychology, Universidad Loyola Andalucía, Dos Hermanas, 41704 Sevilla, Spain; sfernandez@uloyola.es; 2 Department of Psychology and Anthropology, Faculty of Education, University of Extremadura, 06006 Badajoz, Spain; 3 Department of Personality, Assessment and Psychological Treatments, School of Psychology, University of Seville, 41010 Sevilla, Spain; clnunez@us.es

**Keywords:** cannabis, confinement, COVID-19, lockdown, Spain

## Abstract

The impact of the COVID-19 pandemic on our lives is unquestionable, including in the area of substance consumption. This study aimed to evaluate the changes in the pattern of cannabis use during the Spanish COVID-19 lockdown and confinement, and to analyze the variations in the reported motives for cannabis use and withdrawal symptoms. A cross-sectional retrospective study was conducted between April and May 2020, using an instrument that included two time points. Time 1 collected retrospective information on the participants’ habits (N = 89; 73% male; mean age = 29.01) prior to confinement and Time 2 collected the same information during the confinement. Sociodemographic data were collected, as well as the frequency of substance use, cannabis use patterns, sources of cannabis, perceived availability of drugs and cannabis price, and the Marijuana Motives Measure questionnaire and the Cannabis Withdrawal Scale were used. Results showed a decrease in both cannabis use and consumption due to enhancement and social motives. All reported sources of cannabis experienced a reduction except for the Internet, which experienced a significant increase. There was a positive correlation between withdrawal symptoms and coping motives before and during the lockdown. These findings will allow professionals to better develop both prevention and intervention strategies.

## 1. Introduction

The COVID-19 pandemic and the necessary quarantines have had a considerable impact on everyone’s lives. Despite abundant information on the impact of the present and previous pandemics on public health, the economy or other issues [1], no previous information exists on the impact of a pandemic on substance use and substance use disorders (SUD). A report from the European Monitoring Centre on Drugs and Drug Addiction (EMCDDA) concluded that during the initial months of the pandemic there was an overall decline in drug use, particularly for recreational drugs such as cocaine and MDMA, due to the national confinements [2].

However, and for the first time, the COVID-19 pandemic has expanded the focus to the impact of pandemics on our mental health, and considerable research has already been carried out. The main results of previous studies indicate that negative psychological consequences include stress, insomnia, depression, fear, post-traumatic stress symptoms, confusion and anger, as well as unhealthy behaviors such as excessive substance use [3,4,5,6]. Moreover, people with pre-existing medical and psychiatric conditions as well as substance use problems seem to have been at greater risk of adverse psychosocial outcomes [5]. In particular, previous studies have shown that both chronic social isolation and loneliness were significantly higher during the COVID-19 pandemic, which is of great concern given the association of these factors with increased depression and suicidal ideation [7].

Regarding cannabis, Europe’s most commonly used illicit drug [8], results remain unclear. In general, research has mainly focused on alcohol and smoking use during the pandemic, whereas little attention has been paid to cannabis use and abuse [3]. Recent studies indicate that cannabis use increased during the pandemic in various countries [3,9,10,11,12]. In fact, this might have resulted in more emergency department admissions during lockdown compared to the corresponding weeks of 2019 [13].

To date, only a few studies have evaluated the possible factors behind such changes, including the impact of lockdowns on the motives for cannabis use, the perceived availability, withdrawal symptoms, the use of other substances or the sources used to obtain cannabis. Cannabis users who engaged in recommended/mandated self-isolation due to the COVID-19 pandemic were using 20% more cannabis than those who did not [3]. Previous research conducted during non-pandemic periods indicated that motivations for cannabis use are important for understanding cannabis use problems and disorders [14,15,16]. In Belgium, it has been reported that boredom, lack of social contacts, loss of daily structure, desire for a reward after a hard working day, loneliness and conviviality were associated with increased substance use (including cannabis) during lockdown [11]. However, this study provided limited information, due to the lack of a validated instrument, a thorough evaluation, and a specific analysis of cannabis use patterns. Furthermore, some studies also indicated that not only have consumers increased their cannabis use but also the frequency of use, since one-third of users became daily consumers during the lockdown [17]. Moreover, this last study highlighted the fact that changes in the pattern of cannabis use might have had an impact on the use of other substances, such as tobacco, alcohol and benzodiazepines [17]. Regarding changes in cannabis availability, research is also scarce [18]. In Europe, Europol and the EMCDDA [19] reported a shortage of cannabis (resin) as well as some difficulties in accessing the substance in some European countries. In addition, an analysis of three major marketplaces revealed a remarkable increase in cannabis trafficking between January and March 2020 [18,20].

Beyond this preliminary literature regarding the impact of the COVID-19 pandemic on cannabis use in the European context, no published studies have accounted for possible changes in the pattern of cannabis consumption during the lockdown period focusing on frequency, motives for use, availability or withdrawal symptomatology among Spanish participants. The present research aims to address this gap in the literature, especially considering the particularly strict lockdown measures that were enforced in Spain due to the pandemic situation. The unprecedented lockdown was imposed on 14 March until 21 June, 2020. The Spanish authorities undertook exceptional measures based on a mandatory confinement period, when much of the economic activity ceased for several weeks and all non-essential workers were ordered to stay at home, therefore encouraging social isolation. The main goals of this study were (1) to evaluate changes in the pattern of cannabis use in the Spanish population during the confinement associated with the state of alarm in Spain and (2) to evaluate changes in motivations for cannabis use and withdrawal symptoms among cannabis users.

## 2. Materials and Methods

The current research was a cross-sectional retrospective investigation conducted between April and May 2020. During this period, the first declaration of a state of alarm in Spain, entailing a mandatory and strict national confinement, was enforced. The instrument used in the study consisted of two sections, evaluating two different time points. Time 1 collected retrospective information on participants’ habits (i.e., their habits immediately before the state of alarm was enforced), and Time 2 collected information on the current using habits and patterns during the mandatory national confinement. All instruments were computerized and administered online. The battery was disseminated through multiple social platforms and institutional websites. All participants provided their informed consent. This study was approved by the Ethics Committee of Universidad Loyola Andalucía.

### 2.1. Participants

The inclusion criteria were (1) being 18 years of age or older and (2) having used cannabis at least once in the previous 30 days at Time 1. A total of 147 participants provided informed consent and agreed to participate. Overall, 58 questionnaires were discarded as they were incomplete or filled out erratically. The final sample consisted of 89 participants (60.54% of the initial sample) who completed the full survey (73% male; mean age = 29.01, *SD =* 9.26; mean age of onset of cannabis use = 16.14, *SD =* 3.09).

### 2.2. Instruments

Sociodemographic information was collected by means of a questionnaire collecting information on sex, age, age of onset of cannabis use and days passed since the enforcement of the state of alarm and the subsequent national confinement (in Spain, 14 March 2020).

#### 2.2.1. Frequency of Substance Use

The numbers of occasions of cannabis and tobacco use in the previous week were evaluated at Time 1 and 2 with Likert-type response items with the following options: never, 1–2 times, 3–5 times, 6–9 times, 10–19 times, 20–29 times, 30 times or more.

#### 2.2.2. Cannabis Use Patterns

The frequency of use of the different cannabis forms (marijuana only, hashish only, marijuana and tobacco, hashish and tobacco, synthetic cannabinoids, cannabidiol products or other cannabis products) at Time 1 and Time 2 was evaluated with a Likert-type response format including the following answers: never, almost never, sometimes, nearly always, always.

#### 2.2.3. Sources of Cannabis

The frequency of use of different sources of cannabis (friends, relatives, cannabis clubs, Internet or dealers) at Time 1 and Time 2 was evaluated with a Likert-type response format including the following answers: never, almost never, sometimes, nearly always, always.

#### 2.2.4. Perceived Availability of Drugs

The perceived availability of different drugs (marijuana, hashish, other cannabinoid products, tobacco and cocaine) was evaluated at Time 1 and Time 2 with a Likert-type response format with answers ranging from 1 (very difficult or nearly impossible) to 5 (easy or very easy). The questions were extracted from the Spanish survey on drug use among secondary-school-age youths (ESTUDES).

#### 2.2.5. Cannabis Price

Participants were asked about the estimated price they usually paid for the equivalent amount of cannabis necessary to roll a joint at Time 1. Also, they were asked about the price they had paid at the last time of purchase since the declaration of the state of alarm (Time 2). In case they had not paid for cannabis during this time, the hypothesized price they would have to pay was collected. The possible response options included: EUR 0.50, EUR 1, EUR 1.5, EUR 2, EUR 2.5, EUR 3, EUR 4, EUR 5, EUR6 and more than EUR 6.

#### 2.2.6. Cannabis Use Motives

The Spanish version of the Marijuana Motives Measure (MMM) [21,22] was used to evaluate the reasons reported by participants for their cannabis use at Time 1 and Time 2. The MMM is a self-reported questionnaire made up of 25 questions evaluating five different dimensions, namely, social, enhancement, coping, expansion and conformity. Response options have a Likert-type format with the following answers: almost never, sometimes, half of times, most times, nearly always/always. The overall reliability of the Spanish validation was Cronbach’s alpha = 0.86, with the reliability of subscales ranging from 0.64 to 0.83.

#### 2.2.7. Cannabis Withdrawal Symptoms

The Cannabis Withdrawal Scale (CWS) [23] was used to evaluate the severity of cannabis withdrawal at Time 2. The questionnaire was translated and adapted following the corresponding international guidelines [24]. The questionnaire was translated into Spanish by a bilingual researcher and expert on cannabis, and then back translated into English by two native English speakers. Possible inconsistencies were discussed until a final agreement between experts was reached on the final translation. The CWS is made up of 19 items on the prevalence of different symptoms of cannabis withdrawal in the previous 24 h. Responses have a Likert-type format ranging from 0 (not at all) to 10 (extremely). The original version had a reliability of Cronbach’s alpha = 0.91 [23], and the current version a reliability of Cronbach’s alpha = 0.87.

### 2.3. Data Analyses

Firstly, and to analyze the possible changes in the pattern of cannabis use associated with the state of alarm, bivariate analyses were carried out to evaluate differences in cannabis use, cannabis use patterns, perceived availability of drugs and cannabis prices between T1 and T2. In all cases, effect sizes were calculated where possible (phi for categorical variables and Cohen’s *d* for continuous variables). Secondly, bivariate analyses were used to evaluate the association between motives for cannabis use and withdrawal symptoms. More specifically, Pearson correlations were used. In all cases, confidence intervals of 95% were used. Finally, the statistical package used was IBM SPSS Version 26.

## 3. Results

Table 1 reports changes in the pattern of cannabis and tobacco use, as well as in the sources used to obtain cannabis. A significant increase was detected in the percentage of participants abstaining from tobacco use (*p* < 0.001), and significant decreases were reported in the use of all forms of cannabis (*p* < 0.05) except for CBD products, where no significant changes were detected. Significant decreases were detected in the use of most sources of cannabis (*p* < 0.05) except the Internet, which experienced a significant increase (*p* = 0.001). The average price paid for the amount of cannabis necessary to roll a joint also increased significantly. Among cannabis users who had not bought marihuana in the last week at T2, the hypothesized price also increased significantly.

Table 2 reports the changes in the perceived availability of different forms of cannabis as well as other drugs. Significant decreases in perceived availability were detected for all drugs, including tobacco (*p* < 0.05). With regard to the motives for cannabis use, significant decreases were detected in the use of cannabis for enhancement, conformity and social motives (*p* < 0.05).

Finally, Table 3 indicates that a significant correlation existed, both before and during the SA, between cannabis withdrawal symptoms and cannabis use for coping motives (*r* = 0.338, *p* < 0.05 and *r* = 0.327, *p* < 0.05, respectively).

## 4. Discussion

The main goal of this retrospective study was to analyze changes in the pattern of cannabis use in the Spanish population, focusing mainly on the motivations for using cannabis and withdrawal symptoms during the confinement associated with the state of alarm in Spain. To our knowledge, no previous research has analyzed this phenomenon in Spain. Contrary to previous studies [3,9,10,11,12], and on average, no significant changes in the frequency of cannabis or tobacco use were observed among Spanish users during the lockdown, despite a slight overall decrease in the frequency. However, significantly more participants abstained from tobacco use, and 8% began to abstain from cannabis use. In addition, significant decreases were detected in the use of all forms of cannabis, except in the use of CBD products, which remained stable. These results are not surprising given the significant decreases in the perceived availability of all drugs and the significant increases in the cannabis price reported by participants, in line with the difficulties in accessing cannabis previously reported [19].

Accordingly, cannabis users also reported significant changes in the sources used to obtain cannabis, with significant decreases in the use of traditional sources (friends, families, dealers and cannabis clubs). However, significantly more cannabis consumers used the Internet as a source of cannabis or cannabis products, although this figure still represented only 6.7% of participants. This result is consistent with recent studies that highlight the fact that, in the first months of 2020, online cannabis sales increased by 27%, anticipating the negative consequences of the COVID-19 pandemic on social interaction [20,25]. This information represents a relevant issue, considering the fact that, as a consequence of the social isolation due to the pandemic, more cannabis users are becoming familiar with new sources of cannabis supplies [20].

In addition, these difficulties in obtaining cannabis seem to have had an impact also on the motives for cannabis use. Surprisingly, cannabis use for the purposes of enhancement, conformity or social reasons decreased significantly during confinement, while cannabis use for expansion or coping remained stable. Our results also indicate that, both before and during the state of alarm and national confinement, those reporting cannabis use with coping motives experienced significantly more cannabis withdrawal symptoms. This result is important, considering that, traditionally, motivations for cannabis use are a determinant for understanding cannabis use problems and disorders [14,15,16] and, in particular, coping as a motivation for cannabis use represents a marker for increased cannabis use as well as higher risk of problematic use [14,26]. This is in line with other studies highlighting that those individuals relying on cannabis consumption to manage their negative emotions usually show greater cannabis withdrawal symptom severity and less self-efficacy in refraining from using cannabis in emotionally distressing situations, such as the COVID-19 pandemic, where anxiety, depression and stress levels have risen due to national lockdowns [27,28].

Our study has some limitations which mean that the results need to be interpreted with caution. Firstly, our research enrolled more men than women, which could limit the representativeness of the cannabis-using population; nonetheless, reports in Spain have found that males are more likely than females to use cannabis [29]. Secondly, the sample was of moderate size (N = 89). The use of a larger sample could have allowed for the detection of further changes in cannabis use. Finally, in order to analyze the participants’ reasons for consuming cannabis, we used a well-known standardized tool (the Spanish version of the MMM) that evaluates the main motivations for cannabis consumption (i.e., social, enhancement, coping, expansion and conformity reasons). Nonetheless, such a tool does not provide for an exploration of other motivations for cannabis use such as medical concerns, and such information was not collected.

## 5. Conclusions

In conclusion, results from the present study enhance the necessity of investing in resources that support harm reduction strategies and cannabis cessation programs [18], especially in problematic social situations (such as the COVID-19 pandemic) where additional health problems could inadvertently arise, such as increased substance use [3,18]. This information could provide us with useful clinical information on the behavior of cannabis users, not only in possible further COVID outbreaks, but also in common clinical practice.

## Figures and Tables

**Table 1:** Changes in patterns of substance use between Time 1 and Time 2.

	Time 1	Time 2	Statistic	*p*	Effect Size
Weekly tobacco use	4.13	3.82	1.764	0.081	-
Weekly cannabis use	5.09	4.80	1.845	0.068	-
Abstained from tobacco last week	28.1%	37.1%	37.07	<0.001 *	Phi 0.607
Abstained from cannabis last week	0%	8.0%	-	1.000 *	-
Frequency of cannabis use forms					
Marijuana only	2.48	1.97	4.816	<0.001	d = 0.51
Hashish only	1.42	1.21	2.304	0.024	d = 0.24
Marijuana + tobacco	3.62	3.01	4.760	<0.001	d = 0.50
Hashish + tobacco	3.37	2.83	4.217	<0.001	d = 0.45
Vaporized	1.79	1.47	4.693	<0.001	d = 0.50
Spice	1.11	1.04	2.165	0.033	d = 0.23
CBD	1.64	1.52	1.33	0.187	-
Others	1.81	1.43	4.931	<0.001	d = 0.52
Sources					
Friends	67.4%	36.0%	17.701	<0.001	Phi 0.471
Family	20.2%	11.2%	-	<0.001 *	Phi 0.618
Cannabis clubs	56.2%	12.4%	-	0.020 *	Phi 0.263
Internet	4.5%	6.7%	-	0.001 *	-
Dealer	77.5%	46.1%	11.699	0.001	Phi 0.390
EUR/joint (paid before SA—paid during SA)	1.33 (0.99)	1.77 (1.53)	−2.541	0.015	d = 0.39
EUR/joint (paid before SA–hypothesized during SA)	1.55 (1.10)	2.06 (1.51) **	−2.799	0.008	d = 0.45

* Fisher’s Exact Test; ** Hypothesized.

**Table 2:** Changes in perceived availability and motives for cannabis use.

	Time 1	Time 2	t	*p*	Cohen’s d
Perceived availability					
Marijuana	4.39 (1.17)	2.49 (1.50)	10.24	<0.001 **	1.12
Hashish	4.31 (1.15)	2.41 (1.44)	9.44	<0.001 **	1.09
Other cannabis products	3.38 (1.43)	1.79 (1.12)	6.94	<0.001 **	0.93
Tobacco	4.81 (0.74)	4.55 (1.08)	2.41	0.018 *	0.26
Cocaine	3.56 (1.42)	2.24 (1.51)	5.53	<0.001 **	0.86
Motives for cannabis use					
Enhancement	16.35 (3.94)	15.44 (5.30)	2.24	0.028 *	0.26
Conformity	7.25 (1.12)	5.40 (1.87)	14.60	<0.001 *	1.68
Expansion	11.89 (5.42)	11.45 (6.05)	1.17	0.244	-
Coping	9.23 (3.74)	8.59 (4.27)	1.69	0.096	-
Social	10.32 (3.64)	9.07 (3.49)	3.98	<0.001 **	0.45

* *p* < 0.05; ** *p* < 0.001.

**Table 3:** Correlations between cannabis withdrawal symptoms (CWS) and cannabis use motives.

	Time 1					Time 2				
	Enhancement	Conformity	Expansion	Coping	Social	Enhancement	Conformity	Expansion	Coping	Social
CWS	0.161	0.186	0.168	0.338 *	0.166	0.037	0.135	0.056	0.327 *	0.007

^*^p < 0.05.

## Data Availability

The datasets generated for this study are available on request from the corresponding author.
